# Stereoselective Approaches to β-Linked 2-Deoxy Sugars

**DOI:** 10.3390/molecules30071578

**Published:** 2025-04-01

**Authors:** Clay S. Bennett

**Affiliations:** Department of Chemistry, Tufts University, 62 Talbot Ave, Medford, MA 02155, USA; clay.bennett@tufts.edu

**Keywords:** glycosylation, natural products, catalysis, synthesis, stereoselectivity

## Abstract

This review presents a survey of recent developments in the synthesis of β-linked 2-deoxy sugars. Approaches ranging from catalysis to de novo synthesis are described, with a focus on methods developed in the last 10 years. Where relevant, the application of these technologies to synthesis and mechanistic information is discussed. Finally, it concludes with an examination of the scope and limitations of these technologies, as well as examinations about where the field should head next.

## 1. Introduction

**Figure 1:**
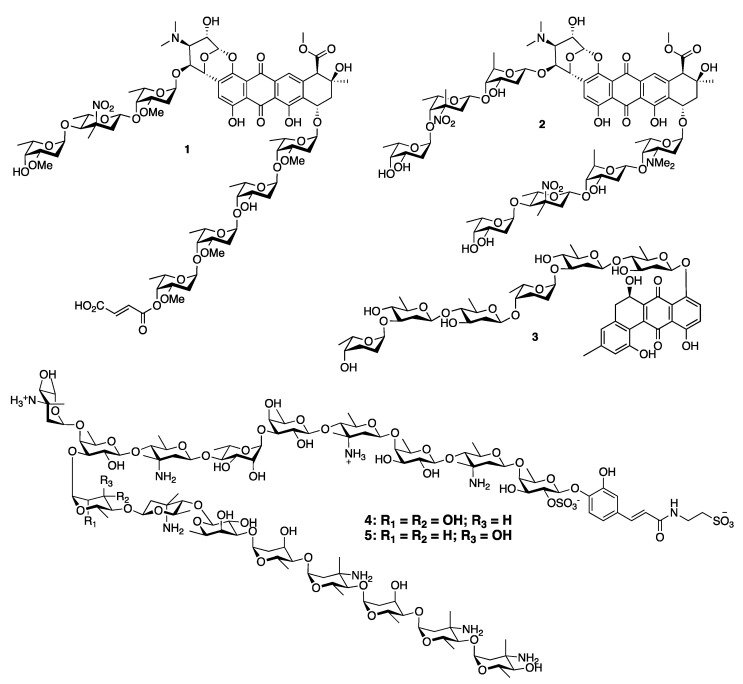
Representative deoxy sugars containing natural products: arugomycin (**1**), keyicin (**2**), landomycin A (**3**), saccharomicin A (**4**), and saccharomicin B (**5**).

Deoxy sugars are a critical component of many natural products, and the composition of these sugars can often play a profound role on biological activity [1]. For example, the nogalamycin natural products arugomycin (**1**) and keyicin (**2**) differ only in the composition of their glycans but possess very different bioactivities (Figure 1). While arugomycin binds to DNA through the major and minor groove [2], keyicin does not interact with nucleic acids [3]. These changes in biological activity with altering sugar compositions are common in nature, leading to the idea that these glycans may be some form of targeting agent to deliver the payload of a natural product to its target. This has in turn led to the concept of glycodiversification, or changing the composition of a natural product’s glycans, as a tool for drug discovery [4,5,6]. While holding enormous potential for therapeutic development, the broader application of glycodiversification, as well as a better understanding of the fundamental interactions between deoxy glycosides and other biomolecules, is hampered by the challenges in producing these molecules.

One of the biggest issues facing deoxy sugar construction lies in controlling the stereochemical outcome of the glycosylation reactions used to link these molecules together. This is the result of a number of factors, including the lack of functionality at the C2 position to install directing groups, the intrinsic stereoelectronic preferences for additions to putative oxocarbenium cations, the instability of many activated deoxy sugar donors, and the limited availability of many deoxy sugar monosaccharides [7,8,9,10,11,12,13]. Owing to many of these factors, the construction of β-linked deoxy sugars is particularly challenging, although for certain substrates that possess an axial C3 substituent, the formation of the α-anomer is kinetically disfavored [14]. This has led to an intense interest in the development of β-specific glycosylation with deoxy sugar donors. While many methods have been developed, their reported scope is often limited to a handful of readily available substrates and may or may not prove general to the staggering diversity of deoxy glycans found in nature [1]. Furthermore, many of the more recently developed methods have yet to be tested in oligosaccharide synthesis, and how they will behave in the context of highly sensitive deoxy sugar oligosaccharides is unknown. After a brief historical perspective, this review will focus on methods that have been developed for the construction of β-linked deoxy sugars in the last decade. This will include the use of remote protecting groups, additives, and catalytic activation. Where relevant, the applications of these methods to the synthesis of deoxy sugar oligosaccharides, as well as the scope and limitations of the methods, will be discussed.

## 2. Classical Approaches to Control Stereochemistry

**Scheme 1:**
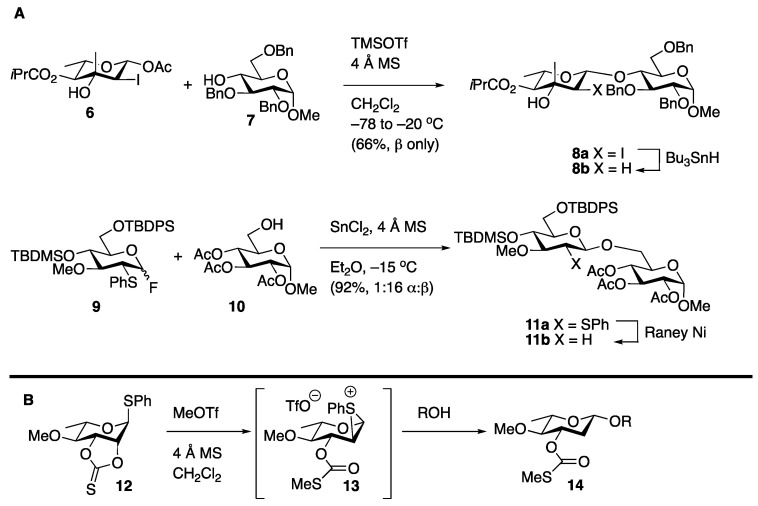
Representative examples of the use of C2 prosthetic groups (**A**) or epi-sulfonium intermediates (**B**) in β-deoxy sugar synthesis.

Classical approaches to β-selective 1,2-*trans* glycosylation reactions in C2-substituted donors employ the use of neighboring group participation [15], which often, although not always [16], proceeds through the intermediacy of a dioxalenium ion (or similar) intermediate to control selectivity. This concept has been frequently extended to β-selective 2-deoxy sugar synthesis through the introduction of a temporary directing group onto the glycosyl donor that could be removed later in the synthesis. These groups have included oxygen atoms for ester attachment [17], C2 halide introduction [18,19], or thioether introduction [20] (Scheme 1A). These groups have met with success in the construction of olivose (2,6-dideoxy glucose)-containing oligosaccharides; for example, the Roush group employed 2-deoxy-2-iodo donors in their synthesis of the landomycin A hexasaccharide [21], and the Yu group took a similar approach in their total synthesis of landomycin A [22]. Alternatively, the use of thioglycosides possessing an activatable group elsewhere on the ring, such as a 2,3-thiocarbonyl, has been employed to provide selectivity through the intermediacy of an epi-sulfonium ion (Scheme 1B) [23], a tactic the Yu group took advantage of in their earlier synthesis of the landomycin A hexasaccharide [24]. These approaches provide products with high levels of selectivity; however, the use of these prosthetic groups necessarily adds additional steps for both their introduction and removal, thereby decreasing the efficiency of the overall synthesis.

**Scheme 2:**
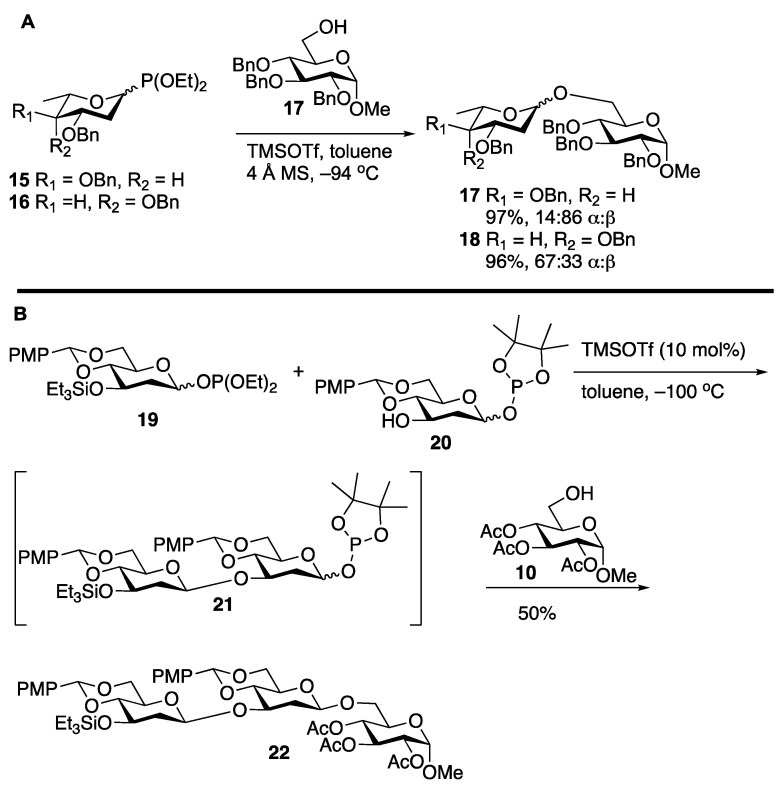
(**A**) Early direct synthesis of β-linked deoxy sugars using glycosyl phosphites; (**B**) applications of glycosyl phosphites to one-pot oligosaccharide synthesis.

In an effort to circumvent these issues, many investigators have attempted to develop direct stereospecific glycosylation reactions for β-linked 2-deoxy sugar synthesis, a tactic that has met with mixed success in early years. In 1995 Hashimoto and co-workers reported that 2-deoxyglycopyranosyl diethyl phosphites underwent β-selective glycosylations upon activation with TMSOTf at −94 °C [25]. Selectivity in the reaction, which presumably proceeded through a glycosyl triflate, was dependent on the configuration of the coupling partners. Olivose donors provided products with moderate to good β-selectivity, while oliose donors did not react selectively (Scheme 2A). In 1998, Sulikowski and co-workers applied this chemistry to the construction of the landomycin A hexasaccharide; however, the authors found that selectivity between complex coupling partners was low at −78 °C, and the authors noted that deoxy glycosyl diethyl phosphites were unstable [26]. These studies did however lead to the observation that it was possible to tune the reactivity of the glycosyl phosphite leaving group to permit one-pot deoxy sugar trisaccharide synthesis (Scheme 2B) [27]. The nature of protecting groups in the activation of deoxy sugar donors can also play a role, and in 2007, Toshima and Takahashi reported that the introduction of sulfonate protecting groups at C3 or C4 of 2-deoxytrichloracetimidate donors permitted selective glycosylation, which was utilized for their synthesis of the hexasaccharide of landomycin A [28,29]. Again, the reaction presumably proceeded through the intermediacy of a 2-deoxy glycosyl triflate, although attempts to detect such species by Crich and co-workers were ultimately unsuccessful [30].

Both the need for the introduction of additional steps using prosthetic groups and the moderate selectivities obtained with direct glycosylation of deoxy sugars under classical conditions present limitations to the use of these chemistries in deoxy sugar oligosaccharide synthesis. This in turn has led investigators to examine alternative approaches where selectivity is not under control of the intrinsic diastereofacial preference of the coupling partners. These approaches include the use of directing groups, catalysis, reagent control, anomeric alkylation/umpolung-based strategies, and de novo-based synthesis.

## 3. Directing Group-Based Approaches

**Scheme 3:**
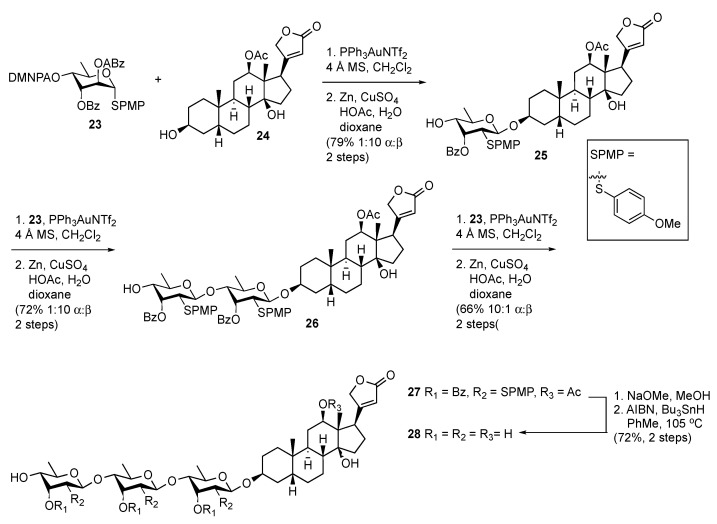
Liao and Sun’s synthesis of digoxin using C-2 ABz-modified thioglycosides.

Recently reported directing group-based approaches most closely mirror classical-based approaches to deoxy sugar synthesis. These approaches can be broadly broken down into two distinct classes. The first approach relies on the use of thioglycosides possessing a leaving group at the 2-position that can be activated under catalytic conditions to generate an epi-sulfonium ion. Mechanistically, the activated leaving group is attacked by the sulfur of the thioglycoside to generate a transient species that is then attacked at the anomeric position to afford β-linked products. Two very recent examples both rely on gold catalysis for the activation of an alkynyl substituent on an axial-configured leaving group. The Liao and Sun groups demonstrated that the activation of *p*-methoxyphenyl manno- or rhamno-configured thioglycosides possessing an *ortho*-alkynylbenzoate (OAbz) group at the 2-position using AuNTf_2_ led to the formation of β-linked 2-deoxy-2-*p*-methoxyphenyl sugars with high levels of selectivity [31]. This chemistry was used for the construction of digitoxin using **23**, possessing a C3 benzoate and C4 2,2,-dimethyl-2-*ortho*-nitrophenyl(acetyl) (DMNPA) protecting groups (Scheme 3) [32,33]. Selectivity in the reactions is high; however, it is hard to dissect how much of the observed selectivity arose through long-range participation of the C3 protecting group [34]. After three iterative rounds of glycosylation, global deprotection and Bu_3_SnH-mediated desulfurization afforded the digitoxin.

**Scheme 4:**
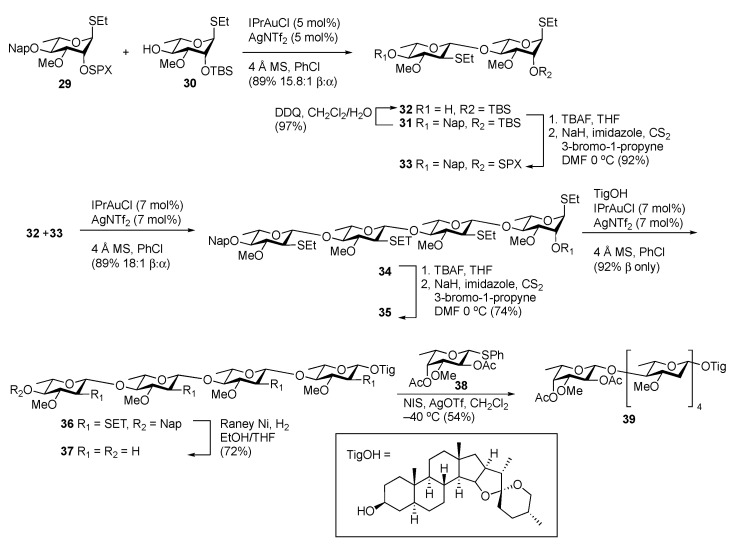
Xu, Liu, and Li’s use of OSPX for β-deoxy oligosaccharide synthesis.

At the same time the Xu, Liu, and Li groups reported that a similar transformation could be affected on donors possessing a C2 *S*-propargyl xanthate (OSPX) leaving group using a combination of IPrAuCl and AgNTf_2_ [35]. In this case, phenyl thioglycoside donors were used, and the authors reported that the OABz leaving group was inferior, highlighting the need to consider both the anomeric and C2 leaving groups when utilizing such tactics. This chemistry was used for the construction of the pentasaccharide from the pregnane glycoside velutinoside A (Scheme 4). After the initial construction of disaccharide **31**, the molecule was split into acceptor **32** and donor **33** through the removal of the Nap group and by converting the OTBS group to the OSPX group, respectively. Coupling of the fragments led to the formation of tetrasaccharide **34** with good yield and selectivity. This fragment could also be assembled through a one-pot [1 + 1 + 2] approach by taking advantage of the orthogonal reactivity of the OSPX and OABz groups to different gold catalysts. Following protecting group manipulations, the coupling of this tetrasaccharide to tigogenin (TigOH), as a model aglycone, desulfurization and Nap removal using Raney Ni, and coupling to fucose thioglycoside **38** afforded the desired target.

In both cases, the authors used DFT calculations to probe the mechanism of the reaction, which supported the idea of an oxocarbenium cation intermediate. Furthermore, Xu and co-workers also reported that they were unable to observe an epi-sulfonium ion intermediate using NMR, similar to reports from Lowary and co-workers, which they used to invoke an oxocarbenium cation-based mechanism [36]. Neither group attempted to look for the putative oxocarbenium cation; however, given that the existence of such species in condensed solution is debated [37], more experimentation is warranted to pin down the exact mechanism of the reaction.

**Scheme 5:**
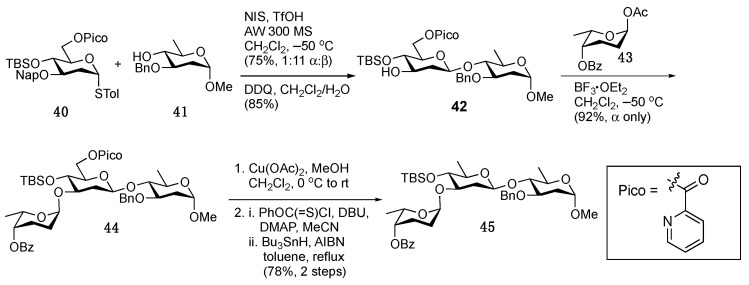
Mong’s synthesis of the landomycin A trisaccharide.

While these approaches do provide access to shorter oligosaccharides, they both also suffer from the limitations of the other indirect approaches described above. While the C2 leaving groups could be installed on readily available coupling partners (such as rhamnose), the reliance on an axial C2 conformation could make the synthesis of other types of glycans lengthy and tedious. An alternative approach relies on the use of directing groups to control selectivity in the reaction. In 2015, the Mong lab took inspiration from the Demchenko group and utilized the picoloyl (Pic) group to construct β-linked 2-deoxy glycosides through hydrogen bond-mediated delivery [38,39]. Through the attachment of this group to the C6 position, the authors were able to obtain high levels of selectivity, which they nicely illustrated through the synthesis of the landomycin E trisaccharide (Scheme 5). The need to place this group at the 6-position did represent one limitation to this approach as it required protecting group removal and subsequent Barton deoxygenation to afford the 2,6-dideoxy sugar-containing product [40].

**Scheme 6:**
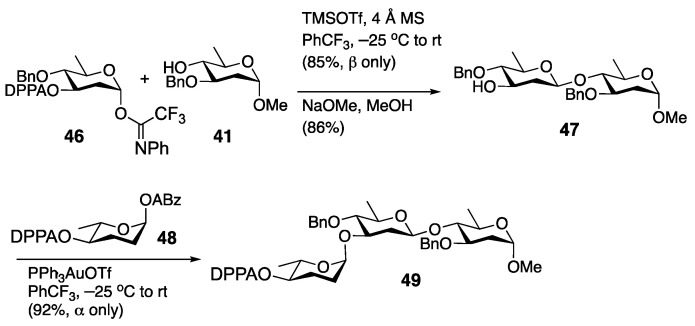
Li’s synthesis of a landomycin E analog using the DPPA directing group.

To address the issue of needing to carry out late-stage deoxygenation, the Li group developed the 2-(diphenylphosphinoyl)acetyl (DPPA) protecting group as an alternative approach for hydrogen bond-mediated delivery. Initially, this group was appended to the C6 position of the donor to afford disaccharides; however, this approach required deoxygenation conditions, similar to those described above [41]. Later the Li group found that this group could also be attached to the C3 position of olivose donors and the C4 position of rhodinose donors [42]. Using this new modification, the group was able to synthesize a protected analog of the trisaccharide fragment form landomycin E using *N*-(phenyl) trifluoroacetimidate and ABz donors (Scheme 6) [43,44]. While the DPPA group could be removed on disaccharide **47** using sodium methoxide, no attempt was made to remove it on the more sensitive trisaccharide **48**. Later, the DPPA group was used by Yu and co-workers to synthesize a library of pregnane monosaccharides [45], although no attempt was made to utilize it in the construction of larger glycans.

## 4. Catalytic Approaches

While directing group-based approaches have clearly proven to be useful for the synthesis of short deoxy sugar oligosaccharides, selectivity in these reactions is highly dependent on the stereochemical configuration of the substituents on the glycosyl donor. To attempt to overcome this constraint, many investigators have developed catalytic approaches to β-linked deoxy sugar synthesis. While glycosylation reactions that are catalytic in the promoter have been known for decades [46], it is only in recent years that catalysts designed to exert control over the stereochemical outcome of the reaction have begun to emerge.

**Scheme 7:**
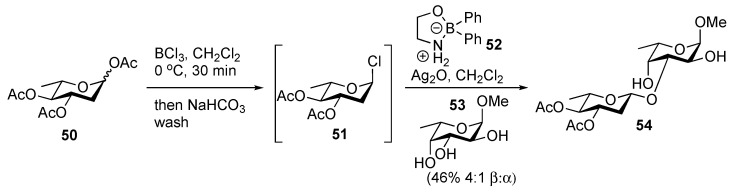
Taylor’s regioselective glycosylation using deoxy sugar chlorides.

An early example of regio- and stereoselective synthesis of β-linked deoxy sugars was reported by the Taylor group in 2014 (Scheme 7) [47]. The group used borinate catalysts, which they demonstrated could be used to selectively acylate the equatorial alcohol of a 1,2-*cis*-diol [48], to regioselectively glycosylate diols and triols with deoxy sugar chloride donors. Owing to the instability of the 2-deoxy glycosyl chlorides, it was necessary to generate them in situ through the treatment of the corresponding glycosyl acetates using BCl_3_ [49]. Treating these species with borinate catalyst **52** in the presence of Ag_2_O and a nucleophile led to glycosylation with excellent regioselectivity. The reactions were moderately β-selective with 2-deoxy sugars being more selective than the corresponding 2,6-dideoxy sugars. Attempts to extend this chemistry to other leaving groups, such as 2-deoxy glycosyl mesylates, failed to provide selective reactions [50].

**Scheme 8:**
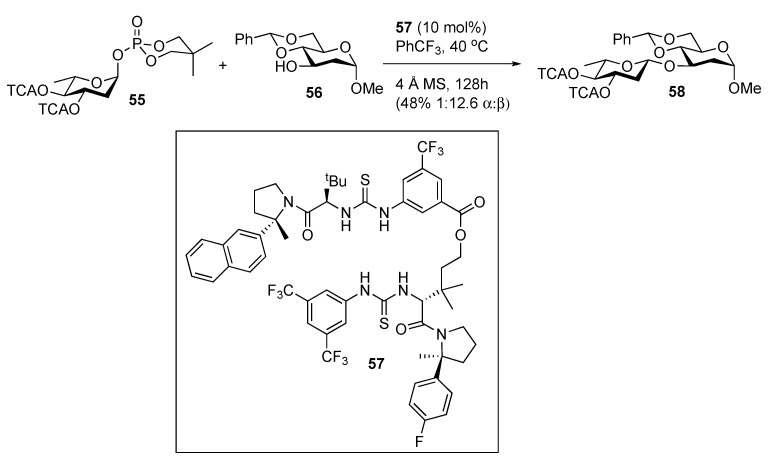
β-Selective glycosylation with Jacobsen’s bis-thiourea catalyst.

Based on previous work involving enantioselective addition to oxocarbenium cations derived from 1-chloroisochromans, in 2017, the Jacobsen group reported that macrocyclic bis-thioureas could activate various glycosyl chlorides for β-selective glycosylation reactions [51]. The catalyst functioned by coordinating with the chloride atom and weakening the C-Cl bond for nucleophilic displacement. Primary ^13^C KIE measurements and DFT calculations indicated that the reactions proceed through an asynchronous cooperative mechanism [52]. Although most of the substrates examined were fully substituted sugars, the group did show that disarmed 2,6-dideoxy sugar chlorides were competent donors in the reaction. Later, in 2024, the group reported that glycosyl phosphates were superior coupling partners in the reaction using a modified catalyst, although for 2,6-dideoxy sugars, it was necessary to use disarming trichloroacetate protecting groups to prevent substrate decomposition (Scheme 8) [53]. As with many catalytic methods, selectivities were better with 2-deoxy sugars than with 2,6-dideoxy sugars, owing to the lower stability of the latter species.

**Scheme 9:**
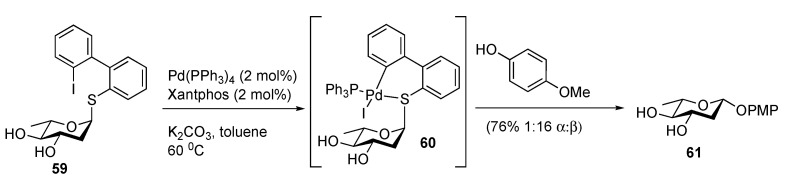
Niu’s Pd-mediated *O*-aryl glycoside formation.

In 2023 Niu and co-workers reported that Pd–Xantphos could activate *ortho*-iodobiphenyl thioglycosides (S*o*IB) for glycosylation with phenolic nucleophiles [54]. The reaction proceeds through inversion of configuration; when the α-S*o*IB anomer is used as a donor in the reaction, the β-product is favored, and the α-product can be obtained from the β-isomer. Notably, because aliphatic alcohols are not reactive under these conditions, unprotected donors can be used in the reaction. Mechanistically, the reaction occurs through the oxidative addition of the aryl iodide to the palladium, which also coordinates the sulfur to give intermediate **60** (Scheme 9). This latter complex can react with the nucleophile through an S_N_2-like manifold to give the product **61**. In support of the mechanism, the authors were able to isolate the palladacycle, obtain its crystal structure, and demonstrate that this intermediate was a competent donor in the reaction. The mechanism was further corroborated through DFT calculations. At present, the reaction is limited to *O*-aryl glycoside formation.

**Scheme 10:**
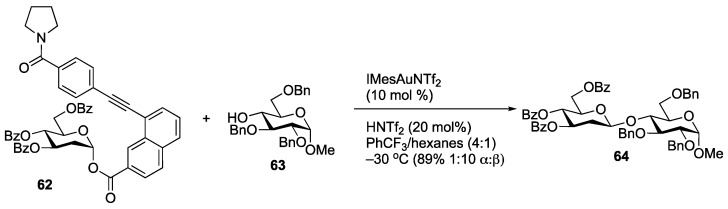
Zhang’s gold-catalyzed glycosylation reaction.

Transition metal catalysis has also been used by Zhang and co-workers, who demonstrated that alkyne-containing 1-naphthoate glycosides, such as **62**, could be activated for glycosylation using catalytic gold (Scheme 10) [55]. The reaction proceeds through inversion of configuration, which the authors attribute to the ability of the amide to hydrogen bond to the incoming nucleophile. Although the major focus of the study was on the construction of 1,2-cis glycosides, the authors did show that benzoate-protected 2-deoxy sugars were competent donors in the reaction. The chemistry was not extended to the more reactive 2,6-dideoxy glycosides commonly found in natural products.

The development of catalytic methods for the activation of donors for β-selective 2-deoxy glycoside synthesis is still a relatively recent development. As a consequence, the scope of most of these methods or their application to total synthesis has yet to be fully explored. Given that the reactivity of different glycosyl donors can span several orders of magnitude [56,57], it is unlikely that a one size fits all method for synthesis will emerge. Rather, it is more likely that tuning of the leaving group/catalyst will be necessary to obtain a broader coverage of the chemical space in selective reactions. This focus on the leaving group (or other intermediates) has led to the development of reagent-controlled methods.

## 5. Reagent-Controlled Approaches

**Scheme 11:**
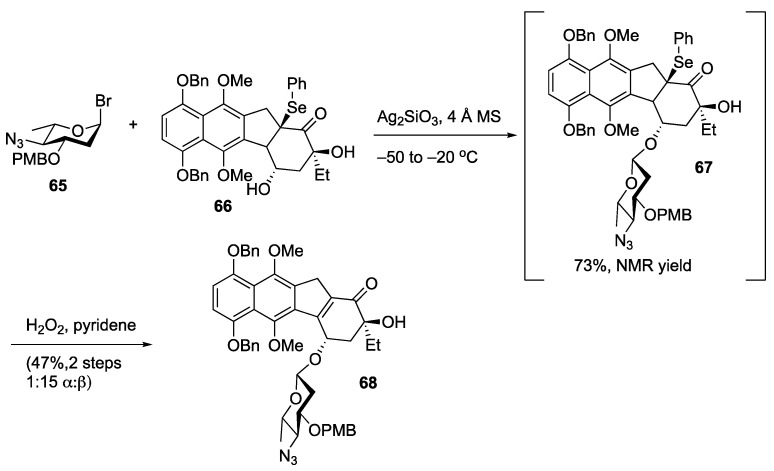
Herzon’s use of glycosyl bromide in the synthesis of lomaiviticin A.

In reagent-controlled approaches, a promoter or additive is added to the reaction with the intent of generating a defined species in situ that can undergo stereoselective reactions. Several modulators have been developed; however, most of these exist for α-selective glycosylations [58,59,60]. When developing glycosylation reactions for β-linked deoxy sugars, the reactivity of both the sugar backbone and the leaving group must be considered. For example, in 2014, Herzon and coworkers studied the stability of several 2-deoxy and 2,6-dideoxy glycosyl bromides (generated in situ from the corresponding glycosyl acetate through treatment with TMSBr) [61]. These studies demonstrated that while armed olivose donors decomposed over 24 h in benzene-d_6_, donors possessing a disarming benzoate group were stable over this time. Furthermore, the authors also found that 2,4,6-trideoxy-4-azido glycosyl bromide could also serve as a competent glycosyl donor, a fact that this group took advantage of in their synthetic studies on lomaiviticin A (Scheme 11) [62,63]. To this end, the activation of donor **65** with silver silicate in the presence of **66** led to the formation of the glycosylated product **67** in 74% yield as a 15:1 (β:α) mixture of anomers, as measured by crude NMR. Because the initially formed glycosylation product was not sufficiently stable for purification, the crude material was subjected to peroxide-mediated conversion to enone **68**, which could be isolated in 47% yield over the two steps.

As an alternative to tuning the reactivity of the sugar backbone, it is also possible to tune the reactivity of the leaving group to obtain stereoselective glycosylation. An example of this was demonstrated by Bennett and co-workers, who demonstrated that activating hemiacetals with tosyl-4-nitroimidazole led to species that underwent β-selective glycosylations with phenolic and thiol nucleophiles [64]. Although aliphatic nucleophiles were poor substrates in the reaction, the group later demonstrated that the activation of the hemiacetals using *p*-toluenesulfonic anhydride permitted the use of metalated aliphatic alcohol nucleophiles in the reaction for β-specific disaccharide formation. Through these studies, the group also demonstrated that the reaction proceeded through the intermediacy of an α-glycosyl tosylate [65]. These intermediates are stable up to −60 °C and presumably react through an S_N_2-like manifold to afford β-linked products.

**Scheme 12:**
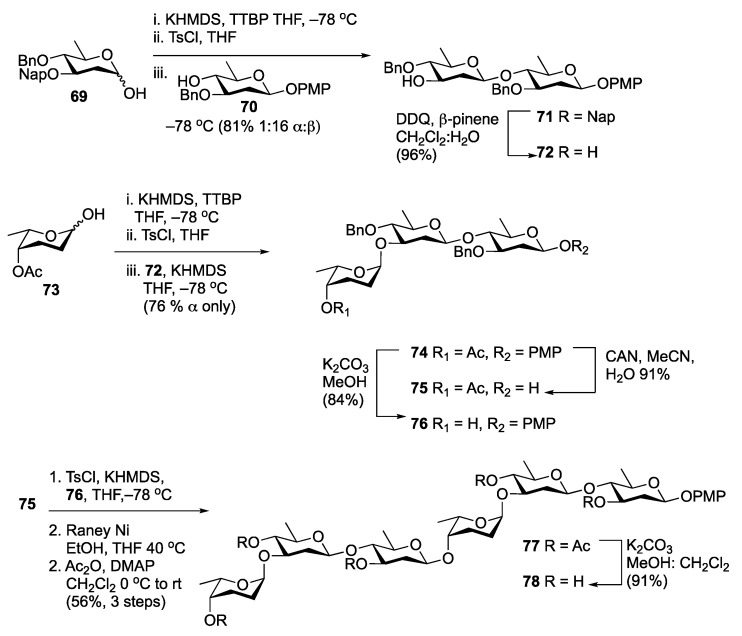
Bennett’s synthesis of the landomycin A hexasaccharide. TTBP = tri-*tert*-butylpyrimidine and DMAP = 4-dimethylaminopyridine.

In an effort to improve the reproducibility and user friendliness of the reaction, the group later reported that *p*-toluenesulfonyl chloride (TsCl) could be used in the reaction [66]. This latter modification permitted the synthesis of several deoxy sugar oligosaccharides, including the trisaccharide from FD-594 and the natural product itself [67], the tetrasaccharide from kigamicin E, the pentasaccharide form saquayamycin Z [68], and the hexasaccharide form landomycin A [69]. The latter synthesis is illustrative of how the process can be applied to oligosaccharide synthesis (Scheme 12). In this process, the activation of donor **69** using TsCl and potassium hexamethyldisilazide (KHMDS), followed by treatment with the potassium alkoxide salt of **70** led to the formation of the disaccharide **71**, which upon 2-naphthylmethyl (Nap) ether removal using 2,3-dichloro-4,5-dicyano-1,4-benzoquinone (DDQ) and β-pinene [70] provided disaccharide acceptor **72**. This compound could be coupled with rhodinose donor **73** to afford trisaccharide **74** as a single α-anomer. The α-selectivity observed in this reaction is an indication that donor **73** is reacting through a different mechanism than olivose donor **69**. Trisaccharide **74** could be then transformed into donor **75** and acceptor **76** through ceric ammonium nitrate (CAN)-mediated removal of the anomeric PMP ether [71] and methanolysis, respectively. The trisaccharide donor and acceptor could be united through another round of glycosylation; however, the perbenzylated product was not stable when exposed to silica. As a result, it was necessary to remove the arming benzyl ethers from the crude hexasaccharide, followed by global acetylation to provide hexasaccharide **77**, which could then be isolated and deacetylated to give the target molecule.

**Scheme 13:**
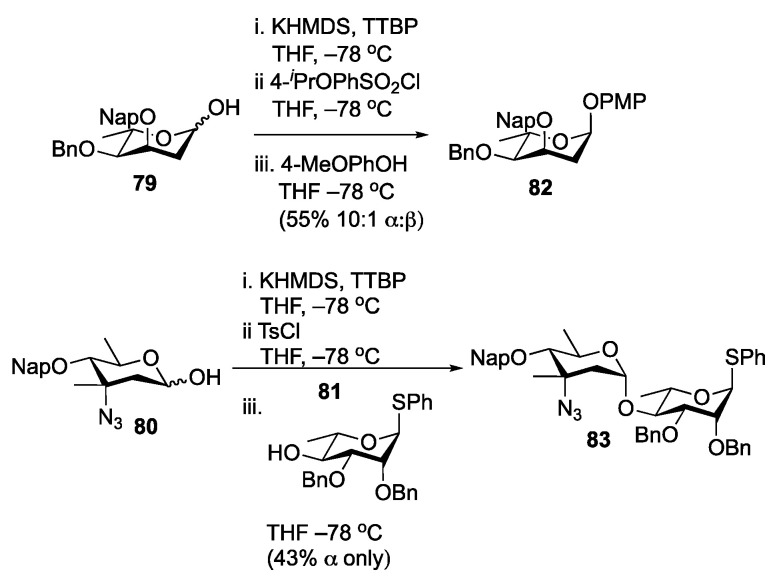
Donors with C3 axial substituents undergo α-selective reactions in sulfonyl chloride-mediated glycosylation.

The α-selectivity observed with the rhodinose donor was an indication that TsCl is not a one-size-fits-all solution for controlling selectivity in glycosylation reactions with deoxy sugars. This was later shown in studies on digitoxose donor **79** and saccharosamine donor **80**, both of which reacted to provide α-linked products upon activation with the electron-rich 4-isopropoxybenzenesulfonyl chloride or TsCl under otherwise identical conditions (Scheme 13) [72,73]. DFT studies on the latter donor indicated that the observed selectivity may be the result of the saccharosamine tosylate adopting a β-configuration in order to minimize unwanted steric and or electronic interactions with the axial C3 azide [74]. Further studies on C2-substituted sugars from the Bennett group indicated that for reactions to be β-selective, the reactivity of the sulfonate leaving group had to be matched to the reactivity of the glycosyl donor, as measured by the donors relative reactivity value (RRV) [75]. As such, with knowledge of the RRV of different deoxy sugars, it should be possible to build up a set of rules for predicting which sulfonate to use with a particular donor. However, these values have yet to be described for most deoxy sugars.

**Scheme 14:**
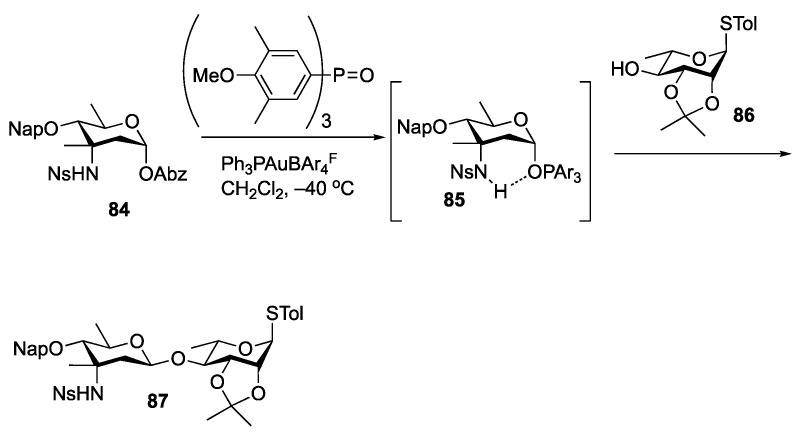
Zeng and Wan’s application of triaryl phosphine oxide modulators for β-selective reactions with C-3 sulfonamide-modified donors.

The presence of axial C-3 substituents can be taken advantage of for the production of β-linked deoxy sugars. An example of this was reported by Zeng, Wan, and coworkers, who demonstrated that while *N*-4-nitobenzenesulfonamide protected saccharosamine donors with an anomeric OABz group underwent α-selective glycosylations upon activation with PPh_3_AuNTf_2_ [76], the addition of tris(3,5-dimethyl-4-methoxyphenyl)phosphine oxide to the reaction resulted in β-specific reactions, including the saccharomicin disaccharide **86** [77] (Scheme 14). The authors posited that selectivity in the reaction is the result of hydrogen bonding between the C3 sulfonamide hydrogen and the oxygen of the phosphine oxide. This latter oxygen also interacts with the α-face of the anomeric carbon of the donor. A nucleophilic attack on the β-face of the molecule then leads to the formation of the product with high levels of selectivity.

The generality of the reactions and the rules for obtaining β-selectivity with reagent-controlled approaches are still not elucidated. The latter chemistries have benefited from a larger examination of the substrate scope, which has helped to establish principles that could be used to make them more general. Importantly, the efficacy of these methods has been demonstrated in total synthesis, which serves as the ultimate test of a method’s utility. However, more work is still needed to understand how the reactivity of different deoxy sugar donors affects the stereochemical outcome of glycosylation reactions. On the other hand, alternative approaches, such as anomeric alkylation and umpolung strategies, have been developed to provide access to β-linked deoxy sugars where the reactivity of the anomeric carbon does not figure into selectivity.

## 6. Anomeric O-Alkylation and Umpolung

**Scheme 15:**
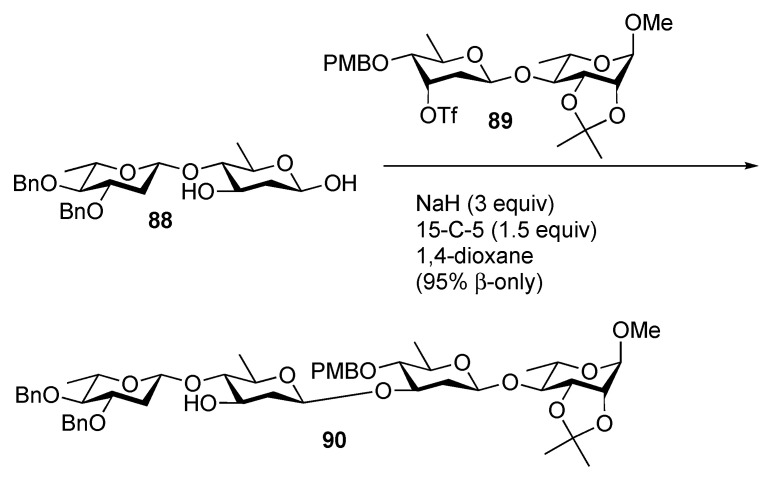
Zhu’s use of anomeric *O*-alkylation for the synthesis of a deoxy sugar tetrasaccharide.

**Scheme 16:**
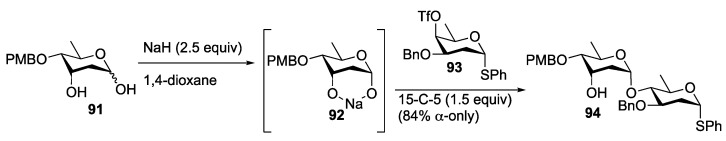
Anomeric *O*-alkylation with axial C3 substituents.

Anomeric alkylation was first reported by Schmidt and co-workers for the alkylation of C2-substituted hemiacetals with simple electrophiles and stereocontrolled trichloroacetimidate formation [78]. In 2004, Shair and co-workers described a report of the alkylation of deoxy sugar hemiacetals with primary and aromatic electrophiles that proceeded with excellent levels of β-selectivity [79]. The reaction worked with primary triflates; however, secondary triflates failed to undergo productive reactions. Later, in 2014, Zhu and coworkers demonstrated that the addition of 15-crown-5 (15-C-5) to the reaction did permit anomeric *O*-alkylation of secondary electrophiles; however, to avoid substrate decomposition, it was necessary to use C3-unprotected sugars, which were also converted to the corresponding bis sodium alkoxide [80]. Regioselectivity in the reaction is the result of repulsion between the lone pairs on the β-linked anomeric alkoxide and the endocyclic pyran ring oxygen, which enhances the reactivity of the anomeric position. Through this methodology, the authors were able to synthesize several deoxy sugar oligosaccharides (Scheme 15), including the trisaccharide from landomycin E [81]. Interestingly, selectivity in the reaction is dependent on the configuration of the C3 substituent. When this substituent is axial, α-linked products are formed exclusively, a factor which the authors attribute to the chelation of the sodium cation between the C1 and C3 alkoxides (Scheme 16) [82].

**Scheme 17:**
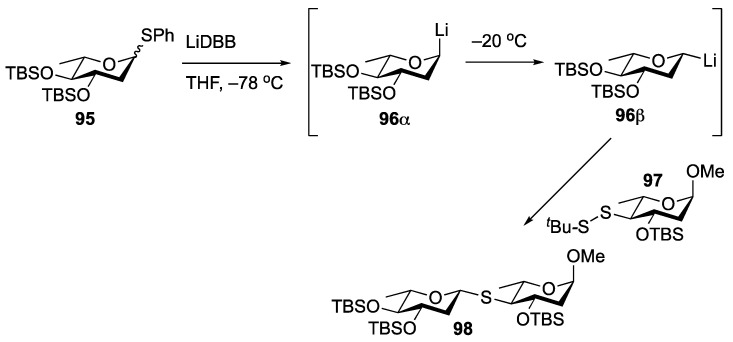
Umpolung-mediated synthesis of *S*-linked disaccharides by Zhu and co-workers.

In 2013, the Zhu lab reported an umpolung approach to *S*-glycoside synthesis based on reductive lithiation of *S*-phenyl glycosides followed by trapping the resulting anomeric anion with *tert*-butydisulfide-containing sugars as the electrophile [83]. After treatment with lithium 4,4′-di-*tert*-butylbiphenyl (LiDBB), the reaction proceeds through the initial formation of an α-lithio species (**96α**), which equilibrates to the corresponding β-linked glycosyl lithium (**96β**) upon warming to −20 °C (Scheme 17). This species can then effectively react at the more sterically accessible position of the disulfide electrophile, leading to the *S*-linked product. The group demonstrated the utility of this chemistry through the construction of an *S*-linked analog of the landomycin A hexasaccharide through a [2 + 2 + 2] process [84].

**Scheme 18:**
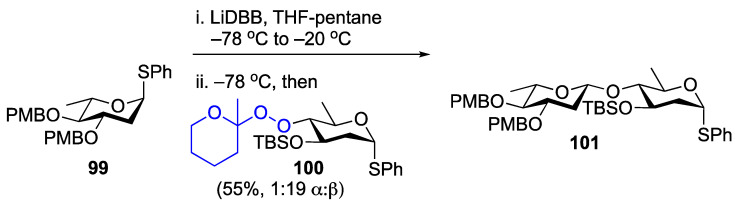
Herzon’s use of MTHP peroxy acetals as electrophiles in umpolung glycosylation. The (2-methyl)-tetrahydropyranyl (MTHP) group is highlighted in blue.

Later, Herzon and coworkers extended this chemistry to *O*-glycoside synthesis using (2-methyl)-tetrahydropyranyl (MTHP) peroxy acetals as the electrophilic component of the reaction [85]. Similar to the work by Zhu, treating olivose and 2-deoxy glucose phenyl thioglycosides with LiDBB resulted in the initial formation of an α-configured glycosyl lithium species, which, upon warming to −20 °C, equilibrated to the β-configured lithium. Cooling the solution back to −78 °C, followed by the addition of the MTHP peroxy acetal leads to the formation of β-linked products with excellent selectivity (Scheme 18). Since the reaction involves pre-activation of the nucleophile, thioglycosides are competent electrophiles in the reaction, permitting one-pot synthetic approaches.

**Scheme 19:**
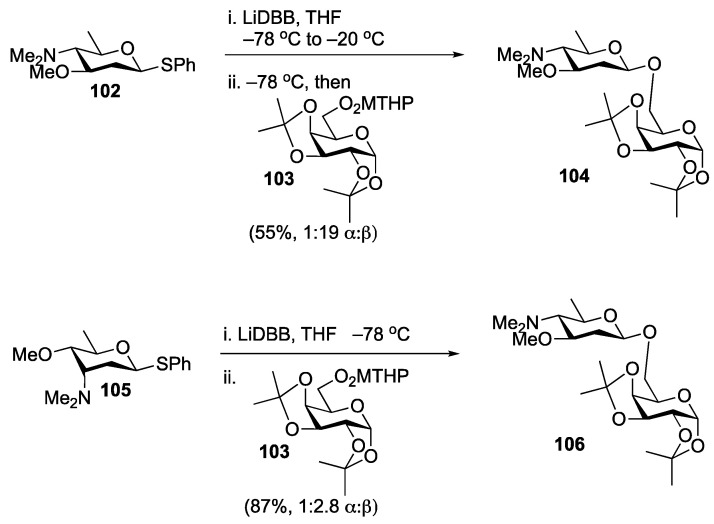
Effect of the amine position and configuration on the stereochemical outcome of umpolung glycosylation.

Subsequent studies of the scope of the reaction demonstrated that dialkyl amines and free hydroxyls were tolerated by the reaction [86,87], although these substrates were more sensitive to substitution. For example, pyrrolosamine derivative **102** reacted with acceptor **103** to provide the disaccharide with excellent selectivity (Scheme 19). On the other hand, the corresponding megalosamine derivative **105** reacted with the same electrophile, without warming to equilibrate to the configuration of the lithium ion, providing **106** with excellent yield and modest β-selectivity. The authors attribute the change in selectivity to the rapid equilibration between the α- and β-radicals formed in **105** upon initial C-S bond cleavage before a second electron is transferred to generate the anomeric anion.

As with other methods, the stereochemical outcome of anomeric *O*-alkylation and umpolung-type glycosylations are highly sensitive to the configuration and functional group content of the “donor”. What works well with 2-deoxy glucose and olivose derivatives can often lead to unexpected results or unselective reactions with other classes of donors. As an alternative, transition metal-mediated de novo synthesis can circumvent some of these issues by forming the glycosidic linkage prior to the introduction of sugar functionality.

## 7. De Novo Approaches

Several approaches to the de novo synthesis of deoxy sugars have been reported in the literature, many of which are directed towards the construction of fully functionalized monosaccharides prior to glycosylation [9]. An alternative approach involves conducting a “glycosylation” reaction on a non-sugar substrate followed by subsequent functional group manipulation to afford the desired target. This approach was pioneered by the O’Doherty group, who demonstrated that 2-*O*-Boc-6-carboxy-2H-pyran-3(6H)-ones could react under Pd(0) catalysis to form acetals with retention of configuration [88]. Subsequent manipulation of the double bond then results in the formation of a deoxy sugar. Since the reaction is stereoretentive, selectivity is entirely dependent on the configuration of the Boc group; hence, β-linked *O*-Boc leads to β-linked deoxy sugars under the proper conditions [89,90].

**Scheme 20:**
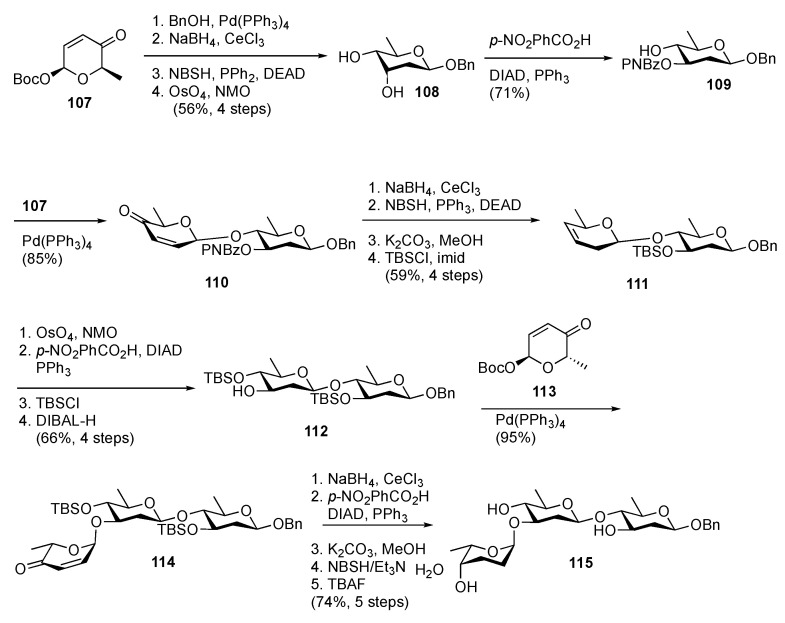
O’Doherty’s synthesis of the landomycin E trisaccharide. NBSH = *o*-nitrobenzenesulfonylhydrazine, DEAD = diethyl azodicarboxylate, and DIAD = diisopropyl azodicarboxylate.

The O’Doherty group’s synthesis of the trisaccharide of landomycin E is illustrative (Scheme 20). Pd-mediated glycosylation of **107**, followed by Luche reduction [91], Myers allylic transposition [92], and dihydroxylation afforded digitoxose **108**. Selective Mitsunobu inversion of the axial C3 alcohol then afforded acceptor **109**. A second round of glycosylation afforded **110**, which was transformed into **111** through a similar sequence. The protecting group swap was carried out in anticipation for a second round of dihydroxylation followed by Mitsunobu inversion and protecting group manipulations to afford disaccharide **112**. A final round of glycosylation using **113** afforded trisaccharide **114**, which could be converted to **115** through standard manipulations.

**Scheme 21:**
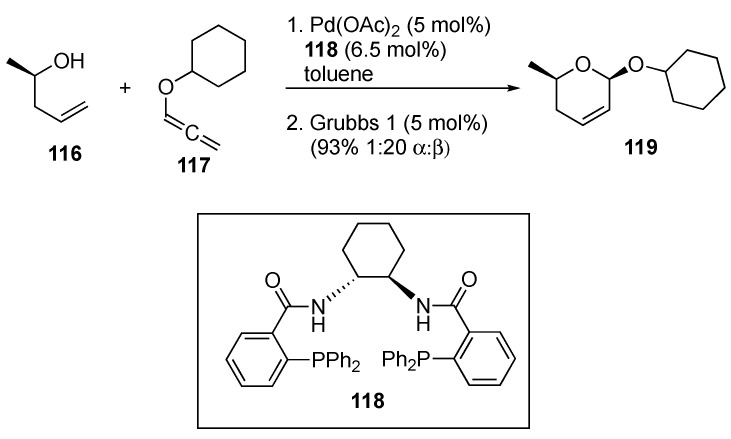
Rhee’s de novo glycosylation strategy.

An alternative approach to de novo synthesis based exclusively on catalyst control was reported by Rhee and co-workers in 2014 [93]. The synthesis involves a two-step process beginning with Pd-mediated coupling between an alcohol and an alkoxy allene (Scheme 21). The resulting ether is then cyclized through ring-closing metathesis. The stereochemistry in the reaction is set by the chirality of the alcohol and the selection of the ligand on the palladium catalysis. The (*R*,*R*) catalyst affords β-linked products with (*R*)-alcohol **116**, while the (*S,S*) catalyst affords the α-linked product. Notably, the use of the enantiomerically pure alcohol affords access to enantiomeric sugars, following post-glycosylation modification.

**Scheme 22:**
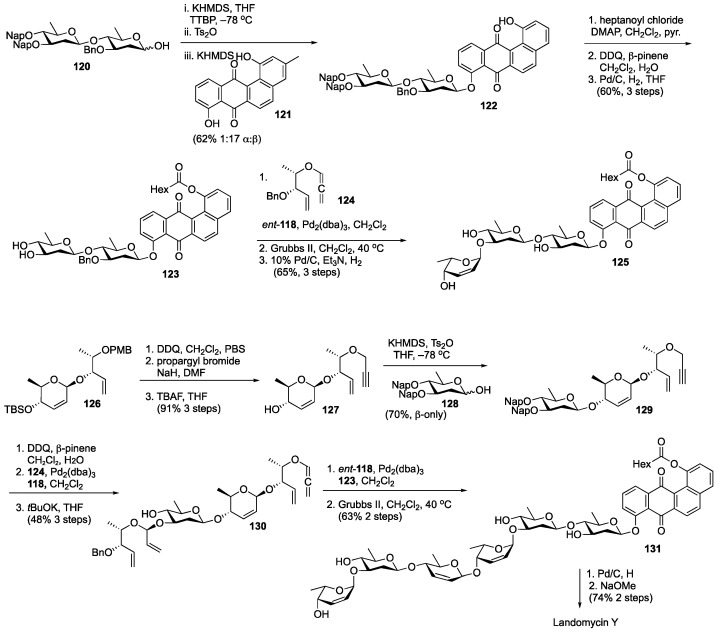
Rhee’s synthesis of landomycin Y.

The Rhee group has used this strategy for the construction of several β-linked deoxy sugar-containing oligosaccharides, including langkocycline oligosaccharides [94], the digitoxin trisaccharide [95], the saccharomicin–rhamnose linkage from saccharomicin [96], and landomycin Y [97]. The synthesis of landomycin Y is illustrated in Scheme 22. Coupling of disaccharide **120** to aglycone **122** under Bennett’s sulfonate-mediated conditions afforded **122** with high β-selectivity. Protecting group manipulations afforded **123**, which was coupled to alkoxy allene **124** via Pd catalysis. The product was subjected to olefin metathesis and hydrogenolysis to afford the trisaccharide landomycin P precursor **125**. To complete the synthesis of landomycin Y, **126** (synthesized through Li’s β-selective Ferrier rearrangement [98]) was converted to propargyl alcohol **127**. Another round of sulfonate glycosylation afforded **129**, which upon subjugation to protecting group manipulations and another round of Pd-mediated glycosylation furnished **130**. This molecule could be coupled to **123** in a subsequent Pd-mediated glycosylation followed by olefin metathesis to afford landomycin Y precursor **131**. Finally, hydrogenolysis of the benzyl ether and olefins, followed by methanolysis of the ester provided the natural product.

These processes nicely illustrate how de novo synthesis, when combined with other approaches, can provide access to 2-deoxy sugar oligosaccharides. The chief limitation of this approach is that olefin manipulation chemistries are not mature enough to provide access to any type of stereochemical configuration of different functional groups (alcohols, amines, and hydrogen) in a single post-glycosylation step. Still, this has not stopped the approach from being applied to very complex molecule synthesis.

## 8. Conclusions

Many different approaches to the synthesis of β-linked deoxy sugars have been developed over the course of the last decade or so, and all of them show promise. In contrast to the well-appreciated differences between the synthesis of a β-glucoside vs. a β-mannoside, developing methods for β-linked deoxy sugar synthesis is often treated as a unified single problem. Unfortunately, deoxy sugars display a wide range of reactivity, and the factors which affect how they behave in glycosylation reactions are poorly understood. As a result, methods that are developed for 2-deoxy glucose, or even olivose, are not likely to provide a general solution to the problem of β-linked deoxy sugar synthesis using a one-size fits-all approach. Instead, a better understanding of how the electronics of different leaving groups, promoter systems, and/or catalysts can be best matched to the reactivity of different sugars is needed to develop reliable models of what conditions are required to forge a particular linkage. A better knowledge of these effects would lead to more rationally designed methods for the construction of these molecules.

In order to achieve this, researchers in the field must begin to address two challenges. The first of which is obtaining a better understanding of the physical organic chemistry governing β-selectivity in deoxy sugar synthesis. While DFT is a useful tool for visualizing a reaction coordinate, it alone is not enough. Researchers need to train such models with kinetics and kinetic isotope effect studies to understand if selective reactions are the result of associative S_N_2-like processes or if other factors are at play. It is likely that such information will reveal that fine tuning of catalysts and/or leaving groups will be necessary to obtain selectivity with different classes of 2-deoxy sugars.

On top of this, studies on new methods for deoxy sugar synthesis must begin to include studies examining the scope of the reaction with multiple classes of deoxy sugar donors. Some groups already do this, and they would acknowledge that one of the biggest bottlenecks to these studies is the production of many deoxy sugar monosaccharides. In the worst cases, some of these donors require ten or more transformations to synthesize from commercial materials using classical methods, which represents a major bottleneck. Hence, there is still a pressing need for the development of new efficient methods to gain access to these molecules so that they can be prepared on a routine basis for both oligosaccharide synthesis and methodological and mechanistic study. Although beyond the scope of this review, there is excellent work being performed in this direction; however, more needs to be done.

With those caveats in mind, there has been excellent work conducted to lay the foundations for more predictable methods to conduct β-selective glycosylations with a range of different deoxy sugar donors. It is now time to branch away from reactions with 2-deoxy glucose, examine the scope of these reactions with different donors, and establish what changes or tweaks need to be made to particular reactions for specific classes of deoxy sugar donors. Given the sheer diversity of deoxy sugar structures, this may sound like a daunting task; however, it also an opportunity to discover new chemistry and develop enabling technologies for glycoscience. In other words, it is a very exciting time to be in the field.

## Data Availability

No new data were created or analyzed in this study. Data sharing is not applicable to this article.
